# A multimodal, risk-stratified framework for AI-driven early risk prediction and personalised prevention in obesity

**DOI:** 10.3389/frai.2026.1865219

**Published:** 2026-06-08

**Authors:** Suning Zhao, Chonin Cheang, Jingyi Lin, Wengioi Mio, Sintong Che, Kaiian Kuok

**Affiliations:** 1 Alfred E. Mann School of Pharmacy and Pharmaceutical Sciences, University of Southern California, Los Angeles, CA, United States; 2 Macau Society for Health Economics, Macao, Macao SAR, China; 3 Macau Yinkui Hospital, Macao, Macao SAR, China; 4 Second Clinical Medical College, Nanjing Medical University, Nanjing, China

**Keywords:** artificial intelligence, digital health, digital twin, dynamic risk score, explainable AI, health equity, just-in-time adaptive intervention, multimodal fusion

## Abstract

Obesity is a multifactorial chronic disease whose worldwide prevalence in adults has more than doubled since 1990, demanding a shift from reactive treatment towards early, personalised prevention. Artificial intelligence (AI) provides a methodological pathway for this shift by integrating heterogeneous, longitudinal evidence—genomic, metabolomic, electronic health record (EHR), wearable Internet-of-Things (IoT), behavioural, and social-environmental—and by translating that evidence into individualised, time-varying risk estimates. Yet the field is fragmented: most existing tools are unimodal, validated on narrow cohorts, opaque to clinicians, and disconnected from the workflows that would render their predictions actionable. In this Perspective we propose an explicit multimodal, risk-stratified framework that links five data layers to a continuous dynamic risk score R(t), defined as a weighted, time-varying aggregation of clinical, anthropometric, behavioural, psychosocial and pharmacological domains. R(t) drives an A/B/C tiering policy that allocates monitoring intensity and intervention modality proportional to risk, and feeds a metabolic–behavioural digital-twin loop in which counterfactual interventions are tested in silico before deployment. We argue that three technical commitments are non-negotiable for translation: (i) cross-modal fusion architectures that respect informative missingness, (ii) explainable, equity-audited risk scoring, and (iii) a five-stage validation pipeline anchored in TRIPOD-AI, decision-curve analysis and post-market drift surveillance. We discuss how this framework reframes long-standing concerns—black-box opacity, demographic bias, real-world fragility—as design constraints rather than afterthoughts, and outline an actionable research agenda for clinically deployable, equitable AI in obesity prevention.

## 1 Introduction

Obesity is a multifactorial chronic disease whose worldwide adult prevalence has more than doubled since 1990. By 2022, an estimated 890 million adults were living with obesity, corresponding to roughly one in eight people globally (World Health Organization, 2024; Hinchliffe et al., 2022). It results from non-linear, dynamic interactions among genetic susceptibility, metabolic dysregulation, behaviour and environment—interactions that traditional assessment tools such as body-mass index (BMI) and lifestyle questionnaires capture only marginally well (An et al., 2022). The clinical consequence is well documented: high-risk individuals are identified late, prevention is reactive, and post-bariatric surgery cohorts suffer substantial attrition, with nationwide French data showing the proportion of patients still attending surgeon-led follow-up falling from 87.1% in the first postoperative year to 29.6% by year five (Hinchliffe et al., 2022; Thereaux et al., 2017).

Artificial intelligence offers a structurally different proposition. By learning from joint distributions of genomic, metabolomic, EHR, wearable, behavioural and environmental data, AI methods can recover non-linear risk surfaces, detect critical inflexion points years in advance, and deliver behavioural recommendations precisely when an individual is most receptive to them (Lee et al., 2025; Forman et al., 2023). Comparative reviews have consistently reported that AI models outperform conventional regression on test data in obesity-related tasks (An et al., 2022; Huang et al., 2025). However, the same body of work also exposes a maturity gap: most studies are unimodal, single-site, retrospective and methodologically opaque (Azmi et al., 2025; Agrawal et al., 2025).

**Figure 1:**
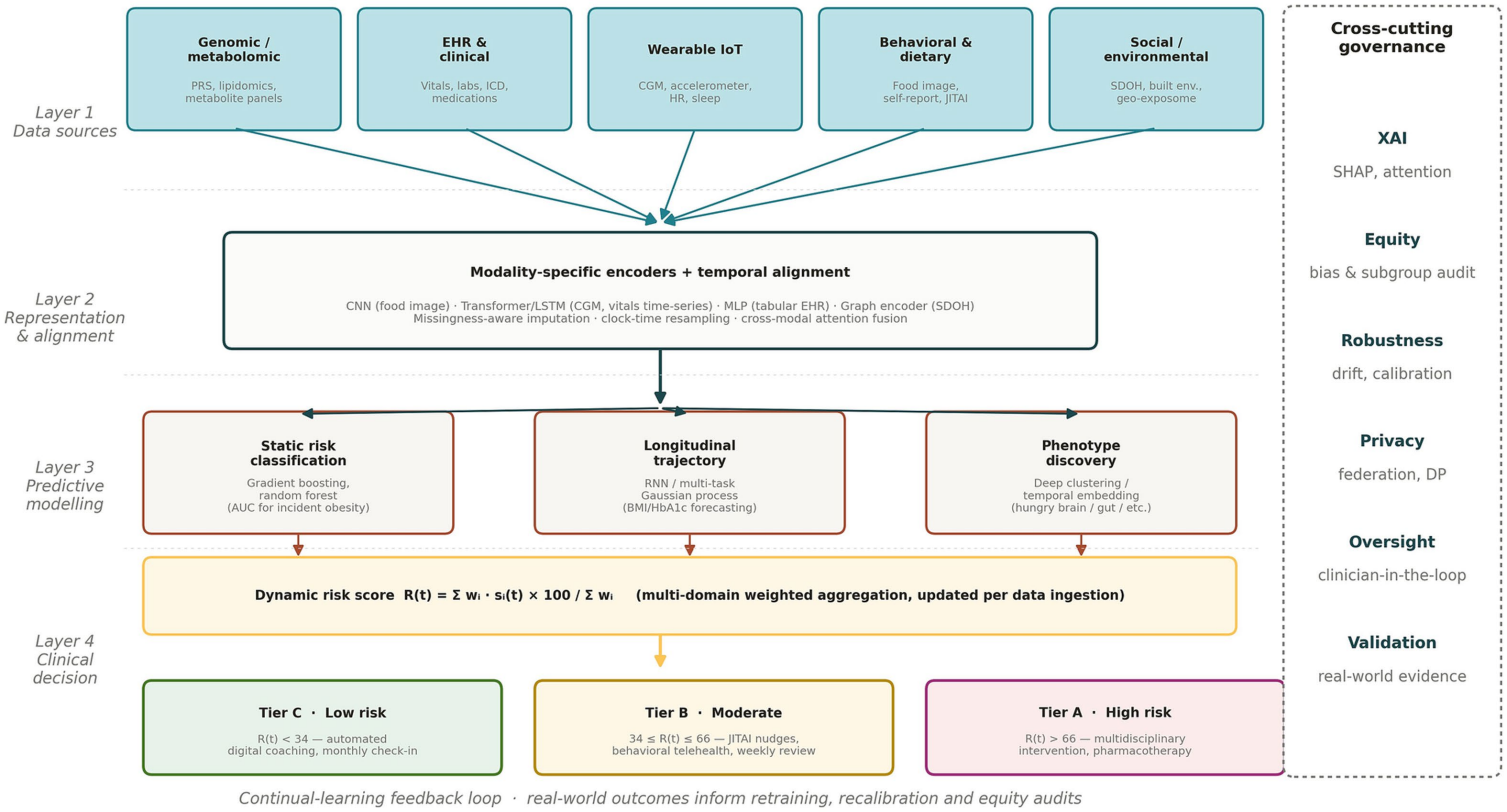
A four-layer, governance-bound architecture for AI-driven obesity prediction and personalised prevention. Genomic, EHR, imaging (DXA, MRI, retinal, CT/US), wearable, behavioural, and social-environmental sources feed modality-specific encoders that are temporally aligned and fused via cross-modal attention; three predictive heads (static-risk classification, longitudinal trajectory, phenotype discovery) feed a continuous dynamic risk score R(*t*) that drives A/B/C-tiered clinical decisions. A cross-cutting governance column—explainability, equity, robustness, privacy, oversight, and real-world validation—binds all four layers and feeds a continual-learning loop that retrains and recalibrates models against deployment-time outcomes.

**Figure 2:**
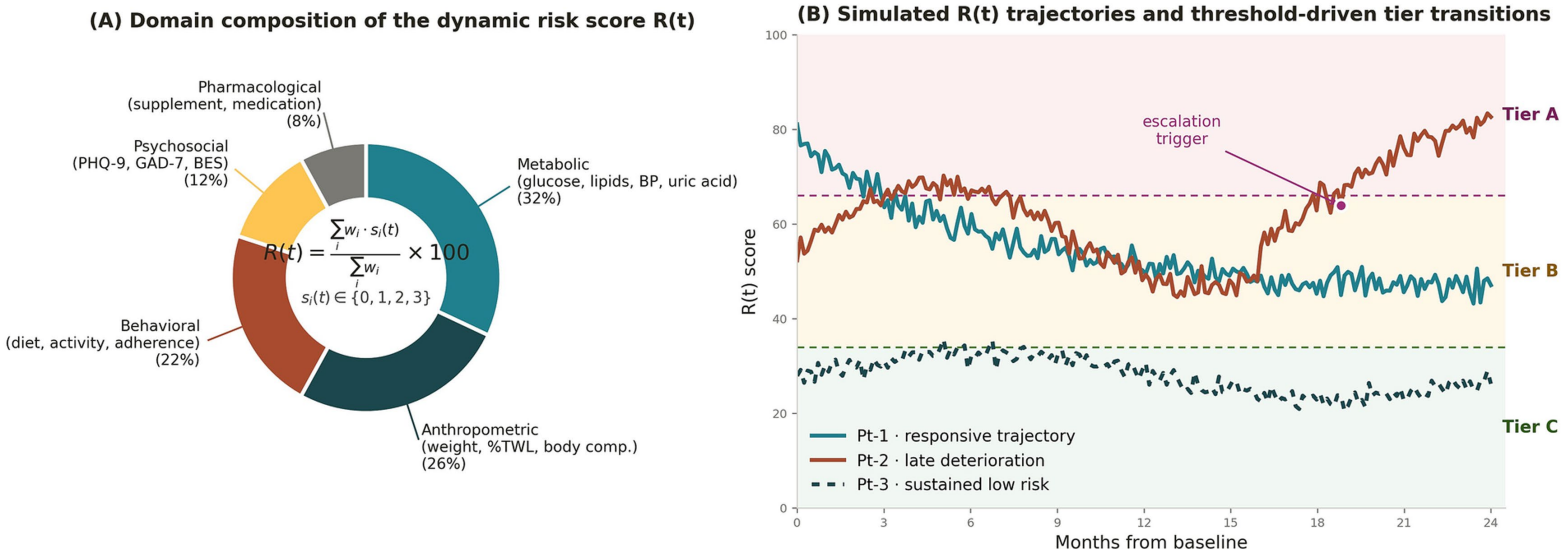
**(A)** Domain composition of the dynamic risk score R(t). The numerical weights shown are proposed, illustrative anchor values, not empirically derived coefficients—they are intended to communicate the relative emphasis we recommend across metabolic, anthropometric, behavioural, psychosocial, and pharmacological domains, and must be re-calibrated against outcome data per population. **(B)** Stylized, simulated R(t) trajectories (not patient data) illustrating how a time-varying score governs longitudinal tier transitions: a responsive trajectory (Pt-1, descending), a late-deteriorating trajectory crossing the Tier-A threshold (Pt-2), and a sustained low-risk trajectory (Pt-3). The red marker indicates an automatic escalation trigger.

**Figure 3:**
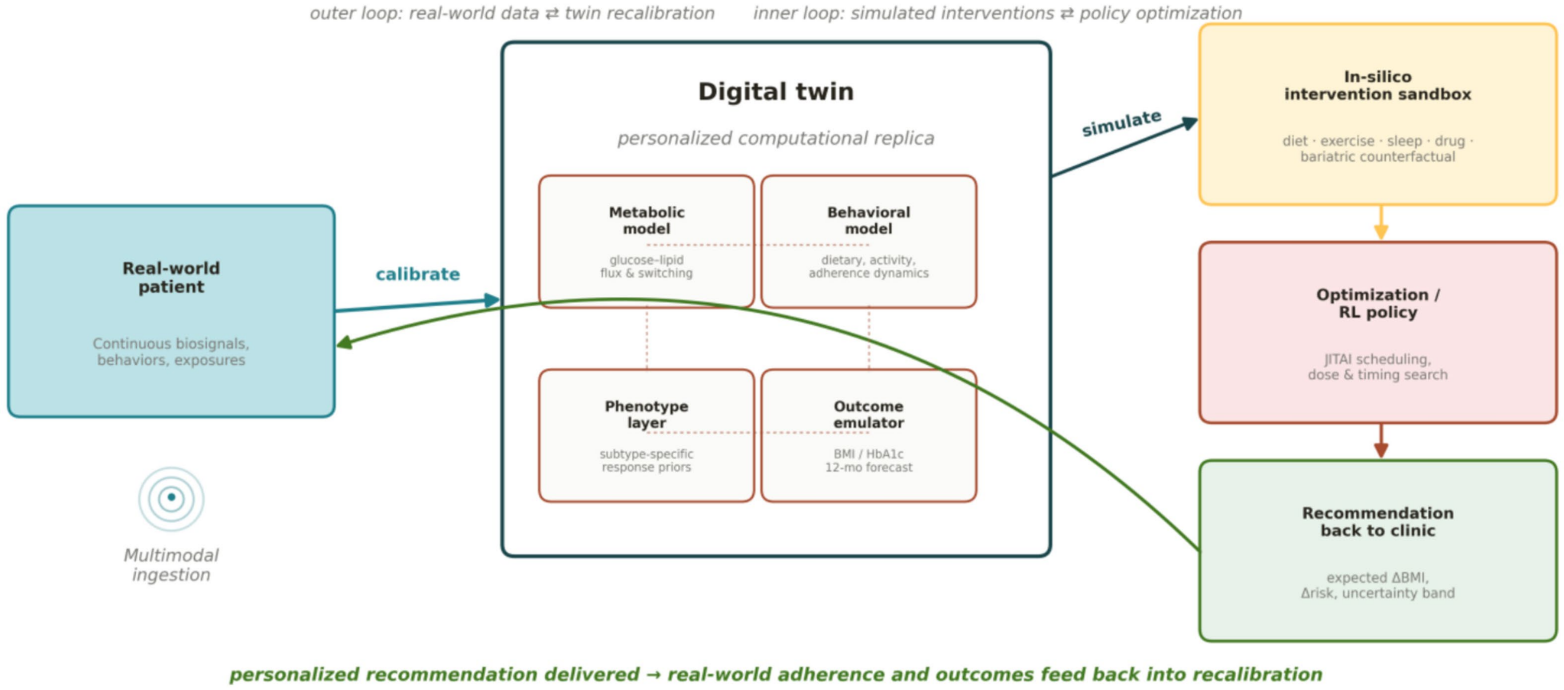
A metabolic–behavioural digital twin closes two loops simultaneously: An outer calibration loop in which real-world signals continuously refine the twin, and an inner optimisation loop in which simulated interventions are evaluated and a reinforcement-learning policy selects an action. The recommendation returned to the clinic carries explicit uncertainty bands, framing the personalisation problem as counterfactual rather than purely predictive.

**Figure 4:**
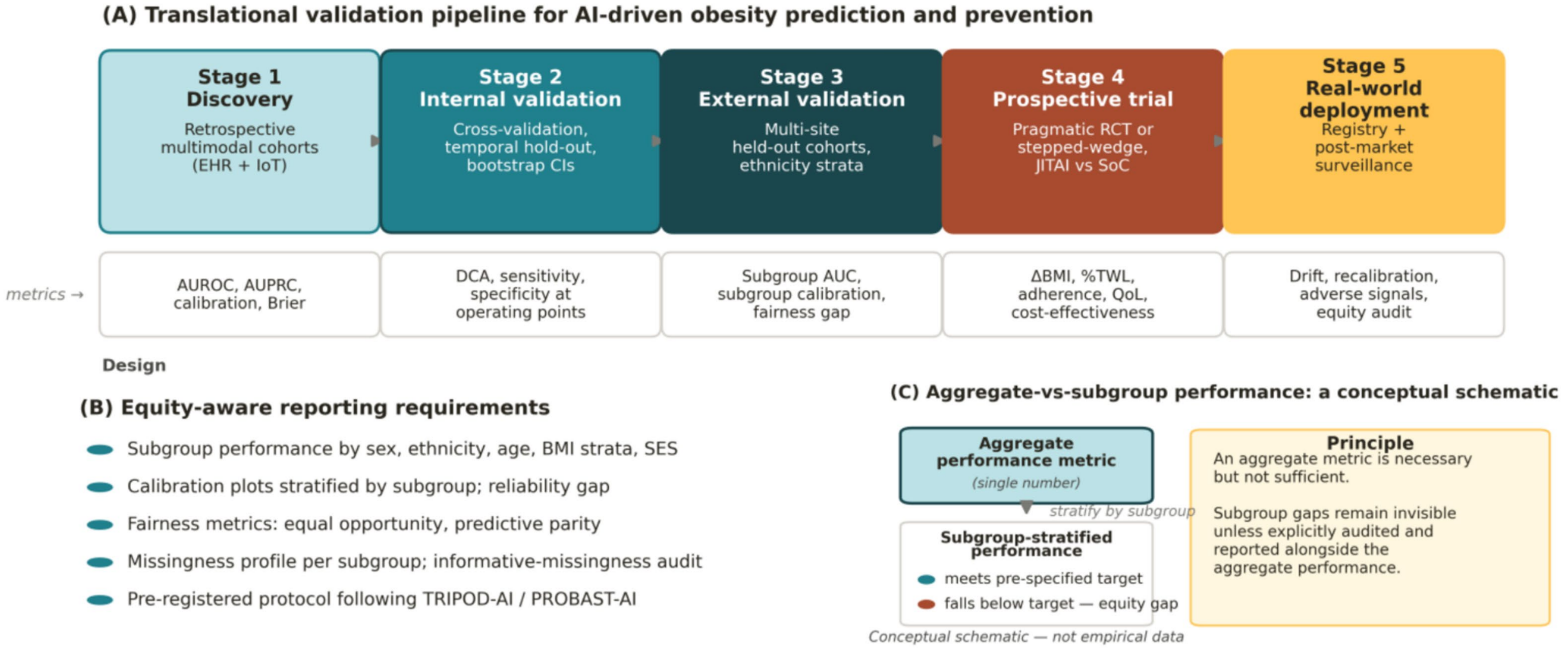
**(A)** Five-stage translational validation pipeline from retrospective discovery through real-world deployment, with stage-appropriate metrics. **(B)** Minimum equity-aware reporting checklist intended for all stages. **(C)** Conceptual schematic of the aggregate-vs-subgroup auditing principle (no empirical values shown): an aggregate metric must be paired with explicit subgroup-stratified reporting to expose performance gaps that single-number summaries conceal.

This Perspective makes three contributions. First, we organise current advances into an explicit four-layer multimodal architecture that distinguishes data ingestion, representation, predictive modelling and clinical decision (Figure 1). Second, we argue that a continuous, time-varying risk score R(t)—rather than static binary classification—is the appropriate quantitative object on which to anchor obesity prevention, and we propose a concrete formulation that operationalises the A/B/C tiring used in chronic-disease management (Figure 2). Third, we extend the digital-twin paradigm into a closed-loop framework that pairs metabolic and behavioural simulation with reinforcement-learning policy optimisation, framing it as a deliberate engineering target rather than an aspirational metaphor (Figure 3). We close by mapping these technical commitments to a translational validation pipeline and to the equity, interpretability and deployment safeguards that, we argue, must be co-designed from the outset rather than retrofitted (Figure 4).

## 2 A multimodal, layered architecture for obesity AI

Recent multimodal studies confirm that synthesising heterogeneous data modalities materially improves risk estimation over unimodal baselines (Lee et al., 2025; Liu et al., 2025). We summarise the resulting design space as four functional layers. Layer 1 ingests five source families: (i) genomic and metabolomic substrates such as polygenic risk scores, lipidomic and metabolite panels; (ii) EHR-derived clinical streams including vitals, laboratory results, ICD-coded diagnoses and medications; (iii) wearable IoT signals such as continuous glucose monitoring (CGM), accelerometry, heart rate and sleep architecture; (iv) behavioural and dietary signals captured through food-image recognition, ecological momentary assessment and just-in-time interaction logs; and (v) social and environmental exposures including social determinants of health (SDOH), built-environment indices and geo-exposome data (An et al., 2022; Hinchliffe et al., 2022; Lee et al., 2025; Carroll et al., 2022).

Imaging biomarkers as Layer-1 inputs. Imaging is integral to Layer 1 phenotyping and to digital-twin calibration. Four modality classes are most informative for obesity: (i) dual-energy X-ray absorptiometry (DXA) for fat-mass index, visceral adipose tissue (VAT) and appendicular lean mass; (ii) abdominal MRI / CT with automated VAT-vs-subcutaneous-adipose-tissue (SAT) segmentation, the current reference for ectopic fat; (iii) hepatic MRI proton-density fat fraction (MRI-PDFF) and controlled-attenuation-parameter (CAP) ultrasound for MASLD staging; and (iv) retinal-fundus deep-learning biomarkers and 2-D/3-D body-shape photogrammetry for low-cost, scalable screening. Imaging-derived biomarkers correct two well-documented limitations of BMI-anchored scoring—they discriminate metabolically-healthy from metabolically-unhealthy obesity at equal BMI, and they re-rank patients whose subcutaneous-dominant adiposity would otherwise be over-prioritised. Concretely, VAT, liver-fat fraction and DXA-derived FMI enter the metabolic and anthropometric domains of R(t) (Table 1) with guideline-informed cut-points (e.g., VAT ≥ 100 cm^2^ in women / ≥ 130 cm^2^ in men; MRI-PDFF ≥ 5% for steatosis, ≥ 10% for moderate-to-severe), and contribute a phenotype prior to the digital twin (Figure 3).

**Table 1:** Indicative multimodal variable map for R(t).

Domain	Example variables	Sampling cadence	Score scale	Indicative weight w;
Metabolic	Fasting / postprandial glucose, HbA1c, LDL/HDL, triglycerides, uric acid, blood pressure	CGM continuous; lab quarterly; BP weekly	0–3	0.32
Anthropometric	%TWL, %EWL, body weight, body-fat %, visceral fat, muscle mass	Daily (smart scale)	0–3	0.26
Behavioural	Caloric intake (image-based), macronutrient distribution, MET-min/week, step count, dietary adherence	Daily	0–3	0.22
Psychosocial	PHQ-9, GAD-7, BES, sleep regularity, social support index	Bi-weekly questionnaire; nightly wearable	0–3	0.12
Pharmacological	GLP-1 RA dose, micronutrient supplementation, medication adherence	Daily log	0–3	0.08

Domains, example variables, sampling cadence, ordinal severity scale and proposed (illustrative) domain weight wᵢ. The weights shown are conceptual anchor values, not empirically derived coefficients; they must be re-fitted to each target population during external validation before clinical use.

Layer 2 reconciles these heterogeneous modalities. Modality-specific encoders—convolutional networks for food imagery, transformers and recurrent networks for CGM and vitals time-series, multi-layer perceptrons for tabular EHR, graph encoders for SDOH and provider networks—produce learned representations that are then aligned in time and fused via cross-modal attention. Two design constraints distinguish a deployment-ready Layer 2 from a published prototype: explicit modelling of informative missingness (devices are abandoned non-randomly, and patients miss visits non-randomly) and clock-time resampling that respects irregular sampling without imputation artefacts (Hinchliffe et al., 2022; Arseniev-Koehler et al., 2025). Without these, downstream predictions inherit and amplify the structure of the missingness rather than the structure of risk.

Layer 3 hosts three complementary predictive heads. A static-risk classifier (typically a gradient-boosted ensemble or shallow neural network) estimates incident-obesity probability from the fused representation; a longitudinal head (recurrent networks, multi-task Gaussian processes, or temporal fusion transformers) forecasts BMI, HbA1c and percentage total weight loss (%TWL) trajectories, even under high missingness (up to 90%; Leroy et al., 2025; Gupta et al., 2022); and a phenotype-discovery head identifies obesity subtypes with distinct treatment responses through deep clustering and temporal embedding (Ruan et al., 2025). The outputs of all three heads converge in a continuous dynamic risk score R(t), described in §3, which serves as the quantitative interface to Layer 4.

Layer 4 closes the loop with risk-stratified clinical decisions and a continual-learning feedback channel. We treat the cross-cutting governance column—explainability, equity audit, drift monitoring, privacy-preserving federation, clinician oversight and real-world validation—not as orthogonal afterthoughts but as first-class design constraints that bind every layer. Table 2 summarises the methodological families currently competing for the modelling slot in this architecture, together with their failure modes.

**Table 2:** Comparative landscape of AI methodologies for obesity prediction.

Methodological family	Representative algorithms	Strengths in obesity context	Limitations / failure modes
Tabular ensemble	XGBoost, LightGBM, random forest	Captures non-linear interactions in EHR + biomarker data; strong baseline; built-in feature importance	Limited handling of irregular time series; risk of feature leakage; calibration drift on new sites
Recurrent / sequence models	LSTM, GRU, Temporal Fusion Transformer	Handles dense IoT streams (CGM, accelerometer); attention reveals influential time windows	Sensitive to missingness patterns; large data requirement; harder to interpret without XAI overlays
Multi-task / joint models	Multi-task GP, shared-encoder MTL	Borrows strength across cohorts; predicts multiple correlated outcomes (BMI, HbA1c, %TWL); robust to high missingness (≤90%)	Specification of shared structure is non-trivial; transfer assumptions must be validated per site
Multimodal foundation / fusion	Cross-modal attention, contrastive pre-training, vision-language models for food intake	Integrates image, text, time-series and tabular data; few-shot adaptation in low-resource subgroups	Compute heavy; opaque latent space; potential to amplify dominant-modality bias
Reinforcement-learning policy	Contextual bandits, offline RL for JITAI	Personalises intervention timing and intensity; reduces coaching workload while preserving outcomes	Reward specification is fragile; safety constraints essential; needs simulator or extensive logged data
Generative / digital-twin	Mechanistic-ML hybrids, neural ODEs	In-silico testing of counterfactual interventions; communicates uncertainty bands to clinicians	Requires domain priors; calibration to individual physiology is data-hungry

Each methodological family is paired with its representative algorithms, distinctive strengths in the obesity context and characteristic failure modes. The right two columns are intended as a deployability check rather than an abstract comparison.

## 3 From static prediction to a dynamic risk score R(t)

A central conceptual move of this Perspective is to anchor obesity prevention not on a static binary classifier but on a continuous, time-varying risk score R(t). Such a score makes three properties explicit: (i) risk is multi-domain and non-substitutable—metabolic deterioration cannot be offset by behavioural improvement of equal magnitude—(ii) risk is temporal, with clinically meaningful inflexion points such as adiposity rebound or post-bariatric weight regain (Leroy et al., 2025; Zheng et al., 2025); and (iii) risk should be auditable at the level of contributing variables rather than only at the level of model outputs.

We adopt a weighted aggregation of domain-specific severity scores:


R(t)=(Σiwi·si(t))/(Σiwi)×100

where each variable i is mapped at time t to an ordinal severity score sᵢ(t) ∈ {0, 1, 2, 3} (0 = within target range; 3 = severe deviation) following pre-specified, guideline-informed cut-points, and wᵢ is a domain-level weight to be empirically calibrated against outcome data. Five domains span the variable space: metabolic (glucose, HbA1c, lipids, blood pressure, and uric acid); anthropometric (%TWL, %EWL, body composition); behavioural (caloric intake, macronutrient distribution, physical activity, dietary adherence); psychosocial (PHQ-9, GAD-7, BES, sleep regularity); and pharmacological (medication and supplementation adherence). The numerical weights shown in Table 1 are illustrative anchor values intended to communicate the relative emphasis we propose for each domain; their final values must be learned per population from outcome data and re-calibrated as deployment evidence accumulates. They are not derived from a published cohort study and should not be interpreted as empirically validated coefficients.

The dynamic score has three operational virtues. First, it is decomposable: clinicians and patients can see which domain is dominating risk at any moment, providing a natural locus for explanation and intervention (Agrawal et al., 2025). Second, it supports threshold-driven escalation: by mapping R(t) onto the A/B/C tier structure used widely in chronic-disease management, the score becomes directly actionable (Figure 2A; Table 3). Third, it integrates naturally with longitudinal models—the trajectory of R(t) (Figure 2B) is itself a clinically meaningful object, with crossings of the Tier-A threshold serving as triggers for escalated review.

**Table 3:** Tier-stratified intervention matrix mapped from R(t).

Tier	R(t) range	Monitoring intensity	AI-mediated intervention	Human-in-the-loop role
Tier C—Low risk	R(t) < 34	Passive collection from wearables; weekly summaries	Automated digital coaching, gamified self-monitoring, monthly AI-generated review	Periodic clinician dashboard review; opt-in escalation
Tier B—Moderate	34 ≤ R(t) ≤ 66	Daily ingestion; weekly check-in; automatic flagging of trend changes	JITAI nudges (RL-optimised), tele-behavioural therapy, dietitian-bot dialogue, targeted PA prescription	Allied health professional review weekly; XAI-supported note generation
Tier A—High risk	R(t) > 66	Real-time monitoring; automatic alerting on threshold crossing	Multidisciplinary intervention plan, pharmacotherapy candidate identification, in-silico counterfactual review	Physician-led decision; AI provides differential and confidence; mandatory override rationale

Each tier specifies monitoring intensity, the AI-mediated intervention modality and the role of the human-in-the-loop. Cut-points are pre-specified rather than implied by model probabilities, allowing transparent governance of escalation rules.

Weight derivation, calibration, optimisation and updating. We propose a three-step procedure. (1) Derivation. Initial weights are learned from a development cohort by fitting a Cox proportional-hazards model with elastic-net regularisation (or, equivalently, a regularised discrete-time survival neural network) in which the outcome is a composite of incident obesity-related complication, weight-regain ≥ 10%, or treatment escalation; normalised hazard ratios become the wᵢ. Bootstrap resampling (≥ 1,000 iterations) provides 95% confidence intervals on every weight, and stability selection rejects domains whose inclusion probability is < 60%. (2) Validation and calibration. Discrimination is assessed by time-dependent AUROC and concordance index; calibration is evaluated by calibration intercept and slope and corrected, where needed, by Platt scaling or isotonic regression before each external deployment so that R(t) values map to the same risk probability across sites. Net benefit at the Tier-B (R = 34) and Tier-A (R = 66) cut-points is checked by decision-curve analysis. (3) Optimisation and online updating. After deployment the weights are refreshed on a rolling window using stochastic gradient updates or Bayesian online learning, with an exponentially-weighted moving average (half-life ≈ 180 days) to balance recency against stability. Two automatic triggers force recalibration: a population-stability-index shift > 0.2 in any input distribution, or a calibration-slope drift outside [0.8, 1.2] over a rolling quarter. Every refresh is versioned, locked, and reported through the post-market surveillance registry described in §6, so that R(t) at any point in time is reproducible and auditable. This procedure aligns with TRIPOD-AI and PROBAST-AI recommendations and addresses the reviewer’s concern about how a time-varying score remains trustworthy as data and populations evolve.

## 4 Personalisation: from JITAI to metabolic–behavioural digital twins

Personalisation in obesity prevention has historically meant tailoring messages to demographic strata. Two complementary advances now permit a stronger interpretation of personalisation. The first is just-in-time adaptive intervention (JITAI): wearable-derived context—location, recent activity, time of day, momentary affect—is used to deliver behavioural prompts precisely when an individual is most likely to act on them, with reinforcement-learning policies modulating both the timing and intensity of contact (Hinchliffe et al., 2022; Forman et al., 2023). Reported gains are non-trivial: comparable weight-loss outcomes have been achieved at roughly one-third of the standard coaching-contact time, with reductions in over-eating, snacking and sedentary periods (Forman et al., 2023; Chew et al., 2024).

The second advance reframes personalisation as in-silico simulation. A metabolic–behavioural digital twin pairs four modules—a metabolic sub-model encoding glucose–lipid flux and fuel switching, a behavioural sub-model encoding dietary, activity and adherence dynamics, a phenotype layer encoding subtype-specific response priors, and an outcome emulator producing 12-month BMI / HbA1c / R(t) forecasts with uncertainty bands (Figure 3). Real-world data from the patient continuously calibrate the twin (outer loop), while interventions—diet, exercise, sleep, drug, or counterfactual bariatric surgery—are simulated against the twin and optimised by a reinforcement-learning policy before any recommendation is delivered to the clinic (inner loop).

Imaging within the digital-twin loop. Within the digital twin, imaging serves three roles. First, baseline DXA / abdominal MRI and MRI-PDFF initialise the phenotype layer (sarcopenic-obesity, VAT-predominant, MASLD-dominant subtypes), determining response priors used by the metabolic and behavioural sub-models. Second, low-cost imaging proxies—body-shape photogrammetry, retinal-fundus biomarkers, bioimpedance—are sampled longitudinally and fed into the outer calibration loop so the twin remains current without repeat high-cost scans. Third, follow-up MRI or DXA at clinically indicated intervals provides ground-truth recalibration anchors that re-fit the twin against real-world body-composition change. This converts imaging from an episodic diagnostic snapshot into a continuous personalisation signal.

Empirical support for digital twins in adjacent metabolic disease is encouraging. In a Cleveland Clinic-led randomised controlled trial of 150 adults with type 2 diabetes, an AI-enabled whole-body digital twin platform achieved a mean HbA1c reduction of 1.3% (vs 0.3% under usual care) and 8.6% body-weight loss (vs 4.6%) at 12 months, with marked de-escalation of glucose-lowering medications (Pantalone et al., 2025). A separate one-year real-world cohort (*n* = 1853) reported a mean HbA1c change of −1.8%, with 89% of participants reaching HbA1c < 7% (Shamanna et al., 2024). Conceptual proposals to extend digital-twin frameworks to broader metabolic-flexibility monitoring further motivate the obesity setting (Su et al., 2025; Bays et al., 2023). Translating this paradigm to obesity prevention has two methodological consequences. First, the recommendation surface delivered to clinicians becomes counterfactual rather than associational—it answers “what is likely to happen if we change diet pattern X by Y %” rather than “is this patient at risk”. Second, uncertainty becomes an explicit output: the twin returns expected ΔBMI, expected Δrisk and a calibrated uncertainty band, supporting risk-aware shared decision-making rather than over-confident point estimates (Senghor et al., 2025).

Practical feasibility, data requirements and deployment pathway. A clinically deployable obesity digital twin can be staged by data-richness. A minimum-viable configuration uses daily smart-scale weight, weekly self-reported food and activity (or smartphone-camera meal capture), quarterly point-of-care HbA1c and lipid panels, and an EHR feed for medications and diagnoses—a stack already available in most chronic-disease programmes. A mid-tier configuration adds continuous glucose monitoring (14-day cycles every quarter), an accelerometer-enabled wearable, and baseline DXA. A research-grade configuration adds abdominal MRI / MRI-PDFF, polygenic risk scores and metabolomics. The metabolic sub-model is computationally light (ordinary-differential-equation glucose-lipid dynamics with personalised parameters fit via Bayesian calibration); the reinforcement-learning policy can be trained offline on logged data and refreshed monthly. Real-world precedents make feasibility concrete rather than speculative: the Twin Health / Cleveland Clinic platform delivered an AI-enabled whole-body digital twin to 150 patients across multiple sites in a 12-month RCT (Pantalone et al., 2025), and a 1853-patient real-world cohort completed twin-guided care with 89% HbA1c remission (Shamanna et al., 2024). A pragmatic deployment timeline is therefore 6–9 months for minimum-viable phenotype + R(t) integration, 12–18 months for outer-loop calibration against site-specific outcomes, and 24 + months for the inner RL-policy loop to mature under post-market drift surveillance. Required infrastructure is summarised in Table 4.

**Table 4:** Stage-specific evaluation metrics across the validation pipeline.

Validation stage	Discrimination & calibration	Clinical utility	Equity & robustness
Internal	AUROC, AUPRC, Brier score, calibration intercept and slope, integrated calibration index	Decision-curve analysis at clinically relevant thresholds; sensitivity/specificity at the R(t) cut-points	Cross-validated subgroup performance; bootstrapped 95% CIs
External / temporal	Held-out site AUROC; recalibration on local prevalence; temporal hold-out to detect dataset shift	Net benefit vs. standard-of-care risk score; misclassification costs	Ethnic / SES / sex subgroup AUROC and calibration; missingness audit
Prospective trial	Pre-registered primary endpoint (e.g., %TWL at 12 mo); pre-specified non-inferiority margins	ΔBMI, %TWL, HbA1c, QoL, adherence, cost per kg lost, ICER	Differential effectiveness across subgroups; safety signals
Real-world deployment	Continuous calibration monitoring; population stability index; concept-drift detection	Time-to-escalation, number-needed-to-monitor, downstream resource use	Periodic equity audits, post-market surveillance, mandatory drift retraining triggers

Each row pairs a validation stage with three families of evaluation: discrimination/calibration, clinical utility, and equity/robustness. The pipeline is intended to be pre-registered, with thresholds and subgroup definitions specified before each stage rather than chosen post-hoc.

### 4.1 Alignment with current clinical guidelines

The proposed framework is intended to complement, not replace, existing obesity-care guidelines. The A/B/C tier policy maps directly onto the four-stage Edmonton Obesity Staging System endorsed by the Obesity Medicine Association (Bays et al., 2023): EOSS 0–1 corresponds to Tier C (digital coaching, annual review), EOSS 2 to Tier B (JITAI plus allied-health support), and EOSS 3–4 to Tier A (multidisciplinary intervention with pharmacotherapy or surgical evaluation). For cardiometabolic risk, R(t)'s metabolic-domain inputs and cut-points are aligned with the 2023 AHA/ACC/ACPM/ADA/AGS chronic-coronary-disease and PREVENT risk equations and with the 2023 ADA Standards of Care, which now position obesity pharmacotherapy as first-line for adults with BMI ≥ 30 kg m^−2^ and at least one weight-related comorbidity. The 2024 ESC guideline on cardiovascular disease prevention and the 2022 AACE algorithm on obesity-related complications inform the metabolic and anthropometric cut-points for tier escalation. WHO 2024 and NICE NG246 supply the public-health and primary-care anchors—universal screening, behavioural-intervention floor, and bariatric referral thresholds—that the digital-twin counterfactual layer is required to respect. In short, the framework supplies a computable, continuously updating instantiation of guideline logic rather than a parallel rule set, allowing clinicians and regulators to trace each tier action back to a recognised, peer-reviewed recommendation.

## 5 Methodological pitfalls and the equity imperative

The architecture in §2–4 sharpens, rather than dissolves, four known challenges. The first is bias and equity. AI obesity tools trained on demographically narrow cohorts systematically under-perform on ethnic, socio-economic and geographic minorities, with BMI-anchored variables in particular embedding well-documented misclassification of body composition across ethnic groups (Joseph, 2025; Senghor et al., 2025). Without an explicit equity-aware design—subgroup performance audits, fairness metrics such as equal opportunity and predictive parity, and proactive efforts to ensure equitable access to digital health technologies—these tools will widen rather than close existing disparities (Hinchliffe et al., 2022; Radanliev, 2025).

The second is data quality. EHRs contain missing values, irregular measurement timing and documentation biases; wearables exhibit sustained non-adherence with informative missingness; and data collected under controlled study conditions may fail to generalise to real-world clinical populations (An et al., 2022; Azmi et al., 2025). The architectural response is a missingness-aware Layer 2 and a fusion layer that reasons about modality availability rather than assuming complete inputs (Hinchliffe et al., 2022).

The third is interpretability. Clinicians require an explanation surface, not a probability (Agrawal et al., 2025; Cangelosi et al., 2025; Hassan et al., 2024). The structure of R(t) makes domain-level decomposition explicit by design; SHAP and attention overlays add per-variable attribution (Agrawal et al., 2025); the digital twin contributes counterfactual explanation. Together these constitute a defensible interpretability stack rather than a single post-hoc explanation tool.

The fourth is integration. Even well-validated models fail when they do not align with EHR interfaces, generate excessive alerts or impose incremental work outside routine workflows (Cangelosi et al., 2025; Hassan et al., 2024). We therefore treat the tier policy and the digital-twin recommendation surface as the AI-clinic interface specification, not as outputs floating beside the workflow. Reimbursement, regulatory classification and liability frameworks are, of course, parallel constraints that the technical architecture must accommodate (Cangelosi et al., 2025).

Implementation barriers in clinical workflows and regulatory practice. Beyond the architectural integration points discussed above, four operational frictions must be planned for. (i) EHR interoperability. Frictionless deployment requires HL7-FHIR R4 resources (Observation, Condition, MedicationStatement, DiagnosticReport) exposed via SMART-on-FHIR launch contexts; vendor-specific deviations and gaps in obesity-relevant LOINC / SNOMED-CT coding are common. (ii) Alert fatigue and cognitive load. JITAI nudges and tier-escalation alerts must be governed by an explicit alert budget, batched daily summaries for Tier-C patients, and threshold-based suppression for spurious deviations—without this, clinician trust collapses regardless of model performance (Hassan et al., 2024; Cangelosi et al., 2025). (iii) Regulatory classification. A risk-stratified obesity AI that drives pharmacotherapy or surgical referral meets the definition of Software as a Medical Device (SaMD) under the FDA Predetermined Change Control Plan framework (2024 final guidance) and would typically be classified as Class IIa under the EU MDR / IVDR, with additional obligations under the EU AI Act’s high-risk-system regime from 2026 onward. The TRIPOD-AI + PROBAST-AI reporting bundle and the post-market drift surveillance registry described in §6 are designed to satisfy these regulatory expectations from the outset rather than retrofit them. (iv) Reimbursement and liability. In the absence of dedicated AI-service CPT or DRG codes, deployment economics depend on bundled remote-physiologic-monitoring (CPT 99453–99,458) and chronic-care-management codes; liability for AI-recommended escalation should be allocated explicitly in the human-in-the-loop policy (Senghor et al., 2025), with mandatory clinician override rationale for Tier-A decisions. Together these constraints reframe deployment not as a software-release event but as a multi-year clinical-operations programme.

## 6 A translational validation pipeline

We argue that the question “does this AI work?” must be replaced by a staged, pre-specified validation pipeline (Figure 4A). Stage 1—discovery—uses retrospective multimodal cohorts to establish AUROC, AUPRC, calibration and Brier scores. Stage 2—internal validation—adds temporal hold-out, bootstrap CIs and decision-curve analysis at clinically meaningful operating points. Stage 3—external validation—moves to multi-site held-out cohorts, with explicit stratification by sex, ethnicity, age, BMI strata and SES, reporting subgroup AUROC and calibration. Stage 4—prospective trial—evaluates the AI-anchored intervention against standard of care under pragmatic or stepped-wedge designs (Forman et al., 2023). Stage 5—real-world deployment—instruments a registry, monitors drift and population-stability indices, and re-trains under pre-specified triggers.

A minimum equity-aware reporting bundle (Figure 4B) underpins every stage: subgroup performance, stratified calibration, fairness metrics, missingness profile, and a pre-registered protocol following TRIPOD-AI and PROBAST-AI. Figure 4C is a deliberately schematic illustration—without numerical values—of a core diagnostic problem: a single aggregate performance metric is necessary but not sufficient, because it can mask materially worse performance in subgroups defined by sex, ethnicity, age, BMI strata or socio-economic status. Without explicit, pre-specified subgroup auditing such gaps remain invisible to aggregate metrics and propagate into deployment, where they translate into unequal clinical benefit (Joseph, 2025; Arseniev-Koehler et al., 2025).

## 7 Outlook

AI offers a credible technical pathway from reactive obesity management to early, personalised prevention, but only under three conditions. First, multimodal fusion must be designed to respect informative missingness and modality availability, not engineered around them. Second, prediction must be expressed as a continuous, decomposable risk score whose threshold-driven action policy is governed transparently by clinicians, not implied by model logits. Third, validation must be staged, equity-audited and continued into post-market surveillance, with drift and recalibration treated as routine operations rather than emergencies. The technical agenda we sketch—multimodal fusion, R(t)-anchored decision policy, metabolic–behavioural digital twins, and a TRIPOD-AI-aligned validation pipeline—is intended as a concrete starting point for implementation rather than as an endpoint.

Realising these commitments requires cross-disciplinary collaboration among data scientists, clinicians, behavioural and implementation scientists, regulators, ethicists and patient advocates. Done well, AI in obesity will function not as a decisive oracle but as a calibrated, auditable, ever-improving tool that returns choice and agency to patients and their care teams—a transformation in kind, not only in performance.

## Data Availability

The original contributions presented in the study are included in the article/supplementary material, further inquiries can be directed to the corresponding author.
